# Metabolic Syndrome among Refugee Women from the West Bank, Palestine: A Cross-Sectional Study

**DOI:** 10.3390/nu10081118

**Published:** 2018-08-18

**Authors:** Salwa G. Massad, Mohammed Khalili, Wahida Karmally, Marwah Abdalla, Umaiyeh Khammash, Gebre-Medhin Mehari, Richard J. Deckelbaum

**Affiliations:** 1 Institute of Human Nutrition, Columbia University, New York, NY 10032, USA; ma2947@columbia.edu (M.A.); rjd20@cumc.columbia.edu (R.J.D.); 2 United Nations Relief and Works Agency for Palestine Refugees in the Near East (UNRWA), Jerusalem 972, Palestine; m.khalili@unrwa.org; 3 Irving Institute for Clinical and Translational Research, Columbia University Medical Center, New York, NY 10032, USA; wk2@cumc.columbia.edu; 4 Division of Cardiology, Department of Medicine, Columbia University Medical Center, New York, NY 10032, USA; 5 Juzoor for Health and Social Development, Ramallah 970, Palestine; ukhammash@juzoor.org; 6 Department of Women’s and Children’s Health, Pediatrics, University Hospital, SE-751 85 Uppsala, Sweden; Mehari.Gebre-Medhin@kbh.uu.se

**Keywords:** Obesity, metabolic syndrome, refugee, women, Palestine

## Abstract

This study was carried out among Palestinian refugee women in the West Bank to provide data on the prevalence of metabolic syndrome (MetS) and its correlates. Data were obtained from a cross-sectional study of 1694 randomly selected refugee women from the United Nations Relief and Works Agency for Palestine Refugees in the Near East (UNRWA) health centers throughout the West Bank during June and July 2010. In this cohort, 30% of the refugee women were overweight, 39% were obese, and 7% were extremely obese. Based on World Health Organization (WHO) criteria, the age-adjusted prevalence of MetS was 19.8%. The results of the binary logistic regression analysis indicated that older age and younger marital age were significantly associated with an increased likelihood of MetS in the women. The high prevalence of obesity and MetS mandates the implementation of national policies for its prevention, notably by initiating large-scale community intervention programs for 5.2 million refugees in Palestine, Jordan, Lebanon, and Syria, to tackle obesity and increase the age at marriage.

## 1. Introduction

The post-2015 development agenda offers an opportunity to ensure a future framework that fully integrates non-communicable diseases (NCDs), which are the leading global determinants of death in adults, and cause more deaths than all other factors combined [1]. About 80% of deaths attributed to NCDs are believed to occur in emerging low- and middle-income countries [2]. In 2010, the World Economic Forum cited NCDs as one of the most important threats to economic development, alongside the current financial crisis, natural disasters, and influenza pandemics [1]. NCDs are also the leading causes of death in the Arab world, particularly in middle- and high-income countries, where is chaemic heart disease is the number one cause of death [3]. As in the rest of the world, NCDs have increased among Palestinian refugees, and are an added burden on the health care system. For example, in 2011 the prevalence of hypertension and diabetes in Palestinian refugees in the West Bank was 17.3% and 12.5%, respectively [4]. From 2001 to 2011, the number of Palestinian refugees affected by NCDs and treated at UNRWA (United Nations Relief and Works Agency for Palestine Refugees in the Near East) health centers in the West Bank, Gaza Strip, Jordan, Lebanon, and Syria was estimated to have increased from about 85,000 to approximately 212,000, with females (61%) bearing the brunt of the conditions [4].

Like NCDs, the prevalence of obesity has also increased worldwide. Obesity rates are estimated to have nearly doubled between 1980 and 2008 [1]. In the Middle East region, obesity rates are higher among women than men [5]. It is well-known that individuals with overall or abdominal obesity have an increased risk of developing metabolic syndrome (MetS) and diabetes mellitus [6]. As obesity rates continue to rise globally, the prevalence of MetS will also increase [7].

Despite the plethora of studies from other regions of the world, there is a paucity of data on MetS in Palestine in general [8,9,10,11,12]. In a small study conducted in the West Bank in 2001 [11], 58% of refugee women met the criteria for metabolic syndrome. However, this study was limited, as it examined women aged 40–65 years selected from only two of the 19 camps in the West Bank. More up-to-date evidence from larger studies is currently lacking. In line with the UNRWA Health Department strategy to collect systematic data on performance and management indicators, the overall aim of this study is to estimate the prevalence of MetS and its components, including obesity in Palestinian female refugees in the West Bank. Based on a review of the factors associated with MetS [6,13,14,15,16], we postulate that MetS increases with age, younger age at marriage, parity, urban living, and lower levels of education.

## 2. Materials and Methods 

### Description of Target Population and Sampling Design

The UNRWA has provided comprehensive primary health care to four generations of Palestine refugees, who lost their homes and means of livelihood as a result of the 1948 conflict. There are 58 camps in five fields of operation: the West Bank, Gaza Strip, Jordan, Lebanon, and Syria. There are 19 camps in the West Bank, with a total of 874,627 registered refugees [17]. Most of the other refugees live in towns and villages in the West Bank. Some camps are located next to major towns, and others are in rural areas. 

We conducted this cross-sectional study in the 42 UNRWA health centers and mobile clinics throughout the West Bank during June and July 2010. We selected all women ≥15 years who reported to all 42 UNRWA primary health care centers and mobile health clinics in the designated time period. We excluded pregnant women and women in the puerperal period (up to 6 weeks following termination of labor). Data were collected during June and July 2010 by a field team of a doctor and 6 nurses.

Women visiting the health centers or mobile clinics on a day designated for data collection were interviewed and their body measurements and blood pressure were taken, and blood samples were collected the following day at the clinic laboratory to examine fasting blood glucose. A mercury sphygmomanometer was used for the measurement of blood pressure in the right arm. The participants rested quietly in a seated position for at least 15 min before the blood pressure measurement. Systolic and diastolic blood pressures were defined as the average of three readings. Using a measuring board, electric balance, and measuring tape, nurses at the clinic recorded the standing height, weight, and waist of each study index twice with light clothing. Waist circumferences measurements were taken with the measuring tape in a horizontal plane, midway between the inferior margin of the ribs and the superior border of the iliac crest. To avoid inter-subjective error, all measurements were taken by the same person. If the difference between the two measurements was more than 10%, a third measurement was obtained. Body mass index (BMI) was calculated as weight in kilograms divided by the square of height in meters, to determine a designation of underweight, normal weight, overweight, and obesity. Data on hypertension and medication for diabetes were extracted from medical records to minimize report bias. Blood was drawn after an overnight fast and analyzed for serum triglycerides, high-density lipoprotein (HDL) cholesterol, and glucose determinations. Biochemical analysis was conducted on fasting plasma samples, all blood analyses being done at the UNRWA laboratory on the day of blood collection. HDL cholesterol and triglyceride (TG) blood levels were measured using the HUMAN kit from Boehringer Mannheim, Mannheim, Germany. The UNRWA Health Department Ethics Committee, Jerusalem, Palestine, approved the study protocol in January 2010. Written informed consent (or assent) was obtained from the participants and the parents of those aged below 18 years.

## 3. Variables

### 3.1. Definition of Metabolic Syndrome 

The definition of MetS is based on WHO diagnostic criteria [18]. For the purpose of comparison with other regional and international data, the prevalence of MetS is also reported based on the National Cholesterol Education Program (NCEP) Adult Treatment Panel III (ATP III) [19]. Direct standardization of prevalence was performed according to the population data published by the United Nations population division, as well as for the “WHO new world population” [20].

### 3.2. World Heath Organization Criteria

WHO criteria for MetS in epidemiologic studies were met if an individual had impaired fasting glucose, defined as a fasting glucose level between 110 and 126 mg/dL; diabetes (fasting glucose level ≥126 mg/dL); or was on diabetes treatment plus two or more of the following abnormalities: blood pressure of ≥140/90 mmHg or taking antihypertensive drugs, serum triglycerides of ≥150 mg/dL or serum HDL cholesterol of <39 mg/dL, or BMI ≥ 30 kg/m^2^ or waist circumference >85 cm.

### 3.3. National Cholesterol Education Program-ATP III 

ATP III criteria for MetS were met if an individual had three or more of the following criteria: waist circumference > 88 cm, fasting plasma glucose (FPG) ≥ 110 mg/dL, blood pressure ≥ 130/85 mmHg or mediation use, serum triglycerides ≥ 150 mg/dL, and serum HDL cholesterol < 50 mg/dL.

### 3.4. Individual and Household Measurements

Data collected on individual factors were age, age at marriage, social status, parity, education, employment, obesity, hypertension, impaired fasting glucose, and hyperlipidemia. The household variable was the type of residence: urban, rural, or camp. Age of participants was determined to the nearest year.

### 3.5. Statistical Analyses

Means, standard deviations for continuous variables, and percentages for categorical variables were used to describe the characteristics of the study sample. All analyses were performed by age. Chi-square tests were used to compare the distribution of metabolic syndrome components among women with and without obesity. MetS prevalence was adjusted to the WHO world standard for the 2000 population by direct standardization [21]. A binary logistic regression analysis was performed using the dichotomous variable MetS (1 = present, 0 = absent). The independent covariates included in the binary logistic regression analysis were age, type of residence, level of education, age at marriage, and parity. In all statistical analyses, *p* values less than 0.05 were considered significant. Statistical analyses were performed using Statistical Package for the Social Sciences (SPSS) v.17 (SPSS Inc., Version 17.0, Chicago, IL, USA).

## 4. Results

Data were collected on 1694 (98%) women visiting the health centers and mobile clinics at the time of data collection, of which 1562 (92%) had complete blood testing. The missing blood tests are for women who did not return for the blood test. The characteristics of the study sample are summarized in Table 1. The mean age was 39 years. Most women were unemployed and had completed high school education or below. Most women were married. Mean marital age was 19.6 years.

### 4.1. Components of Metabolic Syndrome 

Seventy six percent (1282 out of 1692) of the women in the study were overweight/obese (BMI ≥ 25) and 58% (988 out of 1694) of the women had central obesity, with a waist circumference greater than 88 cm (Table 2).

### 4.2. Metabolic Syndrome 

**Figure 1:**
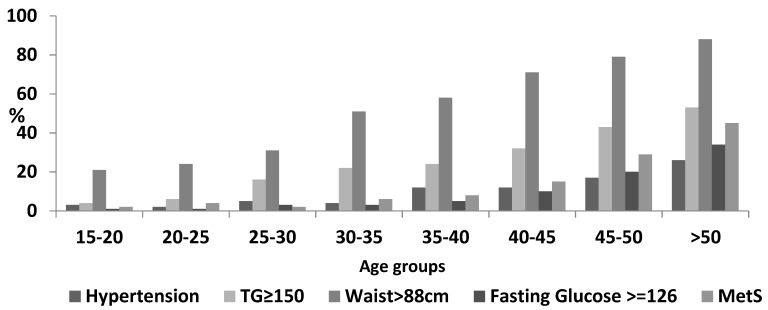
Prevalence of metabolic syndrome and its components by age groups. TG: triglycerides; MetS: metabolic syndrome.

Based on WHO criteria, the prevalence of MetS was 16.9% (Table 2). The age-adjusted prevalence rate based on the WHO standard population was 19.8%. Based on ATP III criteria, 26.6% of women had MetS, with an age adjusted prevalence rate of 28.6%. Similar to the findings on diabetes, hypertension, central obesity, and low HDL, the prevalence of MetS increased significantly with age, with sharp increases occurring after the age of 44 (Figure 1).

The frequencies of the individual components of the metabolic syndrome were more prevalent in obese compared to nonobese women (Table 3). By the ATP III definition, large waist circumference was the most common abnormality in women with obesity. Around 18.4% of obese women and 4% of nonobese ones had four or more components for MetS. A much higher percentage of nonobese (40.9%) than obese women (3.2%) had no MetS component abnormalities. About four times the percentage of obese versus nonobese women had all five metabolic abnormalities indicative of the MetS (4.1% versus 1.1%).

### 4.3. Factors Associated with Metabolic Syndrome in the Study Sample

The results of the binary logistic regression analysis indicate that older age (odds ratio 1.10, 95% CI:1.09–1.12) and younger marital age (1.06, CI: 1.02–1.10) were significantly associated with an increased likelihood of MetS among the women. Based on our sample, the level of education, parity, and residency were not associated with MetS.

## 5. Discussion

We found a high prevalence of metabolic syndrome and its individual components is high in this group. The rate of obesity among Palestinian refugee women 15–64 years in the present study was much higher than that in Palestinian women in the same age group, based on the WHO STEPS survey from 2010–2011 (45% versus 27%, respectively) [22]. Among our cohort, 76% of the refugee women were overweight/obese (BMI > 25), a figure that is higher than in most countries in the Eastern Mediterranean Regional Office (EMRO), apart from Kuwait (79%) [1]. While the majority of those who were obese were aged 45 years and above, rates among adolescent women were also high. The prevalence of overweightness and obesity among adolescent women in our cohort was 38.2%, which is close to that in Kuwait (41.4%) [23]. The UNRWA had provided food rations to refugees living in camps in the form of oil, sugar, rice, flour, and powdered milk, distributed to needy families every three months. These food parcels contributed only a small proportion of food consumption, and the UNRWA replaced food parcels with cash cards in 2016. We believe the main factor behind the high prevalence of obesity is poverty and food insecurity in camps. People consume mostly carbohydrates and fatty foods, rather than fruit and vegetables. This is why the “double burden of under- and overnutrition” remains prevalent in Palestine, among other public health challenges. Based on our earlier published report on a random sample of children aged 6–15 years, 24% of the girls aged 14–15 years were overweight or obese, and 5% had stunting [24].

This high rate of obesity among Palestinian refugee women in this cohort is similar to figures observed in high-income Middle Eastern countries like Kuwait (47.9%), Saudi Arabia (44.0%), and Qatar (45.3%) [25]. The high rate of obesity in our cohort (46.0%) is surprising given the differences in socioeconomic status (SES) between Palestinian refugee women and those in Kuwait, Saudi Arabia, and Qatar. These findings suggest that SES alone does not explain the obesity epidemic, and other risk factors may be present, including biological, genetic, environmental, or nutritional factors that predispose Palestinian refugee women to high rates of obesity. This calls for the identification of local environmental risk factors for obesity, specifically within refugee camps and their surroundings. 

Data on obesity in adult refugee women are limited. A study by Grijalva-Eternod was the first to examine obesity and undernutrition among adults residing for extended periods in refugee settings. Grijalva-Eternod et al. found a similar trend of high obesity rates in a study conducted in Western Saharan refugee camps in Algeria [26]. In that study, the prevalence of obesity among refugee women was 21.9%, a lower figure than that observed in our study population. Additionally, the authors also observed a double burden of obesity and malnutrition among the cohort in Algeria. The possible occurrence of such a status was not assessed in our study.

Only limited data are available regarding MetS in Palestine. Whatever information is available emanates from small studies conducted in the West Bank and Gaza Strip in small geographical areas, or for patients with schizophrenia or cardiovascular diseases [8,10,12,27]. To our knowledge, the present study is the first observational study focusing on obesity and related risk factors among a random sample of Palestinian refugee women in the West Bank. Our results, not previously reported, clearly point to the occurrence of silent yet massive obesity of epidemic proportions among Palestinian refugee women in the West Bank.

Given that Palestinians constitute one of the largest refugee populations in the world, with a population of more than four million distributed across several geographic areas and countries, our findings have important policy implications, both for the local population and globally among adults residing for extended periods in refugee settings. Although further investigations will be required, our findings call for the establishment of a priority NCD focus on obesity, MetS, and diabetes for refugee women. Whether these NCD priorities apply equally to men is a subject for further investigation.

Based on ATP III criteria, the age-adjusted prevalence of MetS among women 20 years old and above was highest in our study sample (27%), in comparison with similar studies among Arab women in Saudi Arabia and Oman, and among Arab-Americans in the United States, where the prevalence was 14%, 23%, and 25%, respectively [15,16,27]. In line with previous studies in Iran [13], Saudi Arabia [15], Oman [28], and Kuwait [6], the prevalence of MetS increased with age. Age-related changes in body size, fat distribution, and insulin sensitivity contribute to the prevalence of this syndrome increasing with age [16]. Overweightness and obesity were associated with MetS. However, the presence of abdominal obesity is more highly correlated with the metabolic risk factors than an elevated BMI (95% of women with MetS had waist size greater than 88 cm), which is in agreement with published reports [29]. Lower marital age was one of the factors associated with MetS in our study sample (11% were married at age 15 or below, and 35% were married before the age of 18). While Sirdah et al., report a higher incidence of MetS among married women, the link between lower marital age and MetS found in our data has not been reported previously in other Palestinian studies [9]. This link may be partially explained by early onset of childbirth, multi-parity, and an increased risk of gestational diabetes; however, these issues were not investigated further in the present study. It is essential to consider the role of non-traditional demographic risk factors, such as marital age and the link to MetS. Direct implications could include the implementation of national policies to eradicate child marriage. 

Although the present study adds to the NCD and MetS literature, it has several limitations. First, this is a clinic-based rather than a population-based study, and was conducted among women attending primary health care clinics. Second, it is a cross-sectional study, and as such, causality could not be examined. Third, our study was limited only to refugee women, and whether refugee men have similar rates of MetS and obesity could not be determined. This study was carried out in one of the five regions covered by the UNRWA, but we believe that the results can be generalized to all refugees in the four remaining regions covered by the UNRWA (Gaza Strip, Lebanon, Syria, Jordan). Further gender-based comparative studies are needed. Despite these limitations, our study is the first to report a high prevalence of MetS and obesity among Palestinian refugee women who reported to all 42 UNRWA health centers and mobile health clinics in the designated time period. 

## 6. Conclusions

Based on our study findings, the high prevalence of obesity and MetS, both of which pose a considerable burden on the middle-aged population, require national policies to be implemented for the prevention, detection, and treatment of these diseases, which are known to contribute to the rising incidence of NCDs. These measures should go hand in hand with holistic, multidisciplinary, and multi-sectorial preventive measures at the individual, community, and societal levels, and should focus on promoting healthy dietary habits and enhancing regular physically active lifestyles, particularly for children and adolescents [30]. Curbing the growing burden of NCDs among Palestinian refugees to reach the “25 by 25” global goals (reduce avoidable mortality from NCDs by 25% by 2025) is a huge challenge for society and the world community in the post-2015 Millennium Development Goals agenda. Management of people with NCDs and multi-morbidity will continue to be an increasingly demanding responsibility for the UNRWA, but is hindered by decreasing resources at its disposal. However, the situation depicted in this report leaves no room for inaction.

## Figures and Tables

**Table 1:** Study sample characteristics (*n* = 1694) for refugee women (15–84 years), West Bank/Palestine 2010.

Variable	Mean (SD ^1^)
Age	38.6 (13.1)
Menarcheal Age	13.6 (1.4)
Age at Marriage	19.6 (4.3)
Marital Status	
Single	15.2%
Married	77.8%
Divorced	1.7%
Widow	5.3%
Number of children	5.4 (3.0)
Number of abortions	1.1 (1.5)
Residency	
City	26.4%
Camp	41.8%
Village	30.3%
Bedouin	1.5%
Work Status	
Employed	13.9%
Unemployed	77.2%
Student	8.9%
Education	
Illiterate	7.9%
Finished up to 12th grade	70.4%
High School Diploma	11.0%
University	10.6%

^1^ Standard deviation.

**Table 2:** Health status of the study sample (*n* = 1694) of refugee women (15–84 years), West Bank/Palestine 2010.

Variable	*n* (% Unless Otherwise Indicated)
Body weight ^1^	
Underweight (Body Mass Index (BMI) < 18.5)	21 (1.2%)
Normal weight (BMI 18.5–24.9)	388 (22.9%)
Overweight and obesity (all)	1282 (75.9%)
Overweight (BMI 25–29.9)	512 (30.3%)
Obesity I (BMI 30–34.9)	422 (24.9%)
Obesity II (BMI 35–39.9)	231 (13.7%)
Extreme Obesity (BMI ≥ 40)	118 (7.0%)
Waist circumference above 88 cm	988 (58.3%)
Low High-density lipoprotein (<50 mg/dL)	445 (28.6%)
Triglycerides *≥* 150 mg/dL	449 (28.8%)
Mean fasting blood glucose level in patients without impaired fasting glucose or diabetes (mg/dL) (SD) ^2^	87.98 (11.20)
Patients with diabetes *n* (%)	193 (11.4%)
On medication *n* (%)	177 (91.7%)
Patients with impaired fasting glucose ^2^	358 (22.9%)
Fasting Blood Glucose (FBG) ≥ 110 mg/dL with/without medication	332 (21.2%)
Normal FBG (FBG < 110 mg/dL) on diabetes treatment	26 (1.7%)
Hypertension (≥140 mm Hg systolic or ≥90 mm Hg diastolic ^3^ and/or take medication)	200 (11.8%)
Metabolic syndrome ^4^	
Based on the definition of the World Health Organization	262 (16.9%)
Based on the definition of The Adult Treatment Panel III	415 (26.6%)

^1^ Data based on 1692 out of 1694 women. ^2^ Estimate based on 1562 out of 1694 women. ^3^ Estimate based on 1692 out of 1694 women. ^4^ Estimate based on 1552 out of 1694 women.

**Table 3:** The relative frequencies of the individual components of the metabolic syndrome and the number of MetS components present in refugee women by body mass index (BMI).

Variable	BMI < 30 kg/m^2^	BMI ≥ 30 kg/m^2^	*p*-Value (BMI < 30 vs ≥ 30 kg/m^2^)
*n* (%)	921 (54.4%)	772 (45.6%)	
Waist circumference > 88 cm	30.2	92	<0.0001
Blood pressure ≥ 130/85 mmHg or medication use	16.0	37.7	<0.0001
Triglycerides ≥ 150 mg/dL	18.6	40.9	<0.0001
Low High Density Lipoprotein (<50 mg/dL)	26.5	31.0	<0.027
Fasting Blood Glucose (≥10 mg/dL) or medication use	12.4	31.6	<0.0001
Number of components			
≥1	59.2	96.8	<0.0001
≥2	27.1	71.6	<0.0001
≥3	12.4	42.5	<0.0001
≥4	4.0	18.4	<0.0001
5	1.1	4.1	<0.0001
